# Production, Characterization and Antioxidant Activity of Electrospun Nanofibers Using Nettle (*Urtica dioica* L.) Seed Mucilage-Polyvinyl Alcohol

**DOI:** 10.3390/polym18151927

**Published:** 2026-08-06

**Authors:** Merve Dağcı Tekin

**Affiliations:** Department of Chemistry and Chemical Processing Technologies, Vocational School of Kütahya Technical Sciences, Kütahya Dumlupınar University, Kütahya 43100, Turkey; merve.dagci@dpu.edu.tr; Tel.: +90-274-443-6213

**Keywords:** electrospinning, nettle seed mucilage, PVA, nanofiber, antioxidant activity

## Abstract

This study aimed to develop naturally derived composite nanofibers by incorporating nettle seed mucilage (NSM) into a polyvinyl alcohol (PVA) matrix using the electrospinning method, and to evaluate their potential as functional bio-based materials. The produced nanofibers were characterized in terms of their morphological, structural and functional properties. The optimized NSM/PVA nanofibers exhibited a smooth, continuous, bead-free and porous morphology with an average fiber diameter of 159.26 nm, indicating the successful production of uniform nanofiber structures. Characterization analyses further confirmed that NSM was effectively integrated into the PVA matrix whilst preserving the composite’s desired structural properties. Furthermore, the incorporation of NSM imparted antioxidant activity to the nanofibers, demonstrating its contribution to the material’s functional performance. Overall, the developed NSM/PVA nanofibers, which combine favorable morphological, structural and functional properties, highlight the potential of stinging nettle seed mucilage as a sustainable natural polymer for the development of bio-based nanofiber composites with potential applications in biomedical coatings, controlled drug delivery, filtration and advanced surface engineering.

## 1. Introduction

Advances in nanotechnology have made it possible to develop nanofibers with high surface-to-volume ratios, controlled morphological properties, and potential applications in many fields such as biomedicine, food packaging, and tissue engineering [1,2]. Electrospinning is a simple, effective, and flexible method widely preferred in nanofiber production, based on the principle of applying a high-voltage electric field to a polymer solution. In this process, the polymer jet emerging from the Taylor cone formed by the effect of electrical forces elongates and solidifies to form a fiber structure as the solvent is removed. The morphology of the resulting nanofibers is significantly affected by process parameters such as applied voltage, feed rate, and distance between the needle and the collector, as well as properties such as solution viscosity, electrical conductivity, and polymer concentration. Therefore, these parameters need to be appropriately optimized to obtain homogeneous and bead-free fiber structures [3,4,5].

Polyvinyl alcohol (PVA) forms an important polymeric carrier matrix frequently used in nanofiber production due to its water-soluble and biocompatible structure and suitability for electrospinning [6]. However, the low biological activity and high-water sensitivity of pure PVA systems can limit their application areas. Therefore, combining PVA with natural polysaccharides, mucilages, or various bioactive components to improve its mechanical, functional, and biological properties is a common approach [7,8]. Plant-derived mucilages are natural hydrocolloids rich in polysaccharides and have a hydrophilic structure. However, due to their low degree of chain entanglement and insufficient viscosity, they may have difficulty achieving the desired fiber formation by electrospinning alone. On the other hand, as shown in previous mucilage/PVA electrospinning studies summarized in Table 1. mixtures with PVA make it possible to produce continuous, stable, and functional composite nanofibers [9,10,11].

Nettle (*Urtica dioica* L.) is a medicinal herb traditionally valued for its antioxidant capacity and the phenolic compounds, flavonoids, and other bioactive substances it contains [12,13]. Furthermore, its functional properties as a food component are well-defined, and its seeds are reported to contain high amounts of oil, protein, and phenolic compounds [14,15]. Nettle seed mucilage is a hydrophilic, polysaccharide-based natural hydrocolloid. Compositional analyses show that this structure consists mainly of carbohydrates (63–77%) and contains smaller fractions of protein, ash, moisture, and uronic acid [16,17]. Its chemical structure, rich in uronic acid groups and hydroxyl groups, supports hydrogen bond formation with PVA chains, making it suitable for use as a functional component in composite nanofiber systems. Therefore, nettle seed mucilage can be considered a natural additive that can impart antioxidant properties to the resulting nanofiber structure [18,19].

This study aimed to produce nanofibers using PVA and nettle seed mucilage via electrospinning and to characterize the resulting structures. The morphological properties of the produced nanofibers were evaluated using SEM analysis, functional group interactions using FTIR spectroscopy, crystal structure changes using XRD analysis, and antioxidant activities using the DPPH radical scavenging test. Thus, the usability of nettle seed mucilage as a natural, functional, and bioactive component in PVA-based electrospun nanofiber systems was investigated.

## 2. Materials and Methods

### 2.1. Extraction of Nettle Seed Mucilage (NSM) and Preparation of PVA Solution

**Figure 1:**
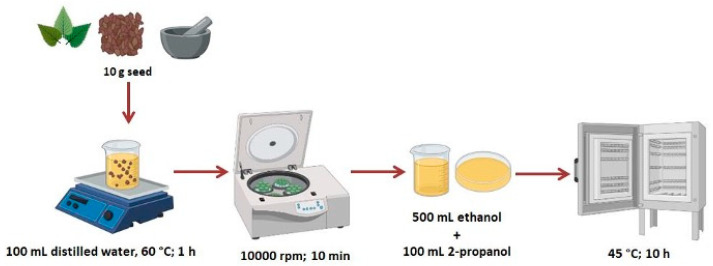
Schematic representation of the process of extracting nettle seed mucilage.

Seeds of stinging nettle (*Urtica dioica* L.) were obtained from the local herbal market in Kütahya, Türkiye in March 2025. Then, 10 g of seeds were ground in an agate mortar. A total of 100 mL of ultrapure water (Millipore Direct-Q3UV, Merck Millipore, Burlington, MA, USA) was added to the ground seeds. It was mixed in a heated magnetic stirrer (WiseStir, MSH-20A, Daihan Scientific Co., Ltd., Wonju-si, Republic of Korea) at 60 °C for 1 h. Then it was centrifuged at 1000 rpm for 10 min (Sigma, 3-30KS, Sigma Laborzentrifugen GmbH, Osterode am Harz, Germany). It was filtered using a fine-mesh cloth to remove impurities. The mixture was left at room temperature for 24 h. The mucilage was transferred to a beaker containing 500 mL of ethanol (Merck, Darmstadt, Germany) and 100 mL of 2-propanol (Merck, Darmstadt, Germany). Then, they were placed in glass Petri dishes and dried in the oven at 45 °C for 10 h [10]. The overall extraction workflow, from raw seeds to dried mucilage powder, is summarized schematically in Figure 1. The dried mucilage was stored at room temperature. To prepare 2% NSM solution, 0.1 g of dried NSM was mixed with 5 mL of ultrapure water/acetone (Merck, Darmstadt, Germany) (70:30) solution on a magnetic stirrer for 2 h.

To prepare 10% PVA (Across Organics, Geel, Belgium; Mw 50,000–85,000 g/mol) solution, 10 g of PVA was mixed in 100 mL of ultrapure water on a magnetic stirrer for 2 h at 80 °C. It was stored at room temperature.

### 2.2. Production of Electrospun NSM/PVA Nanofibers

**Figure 2:**
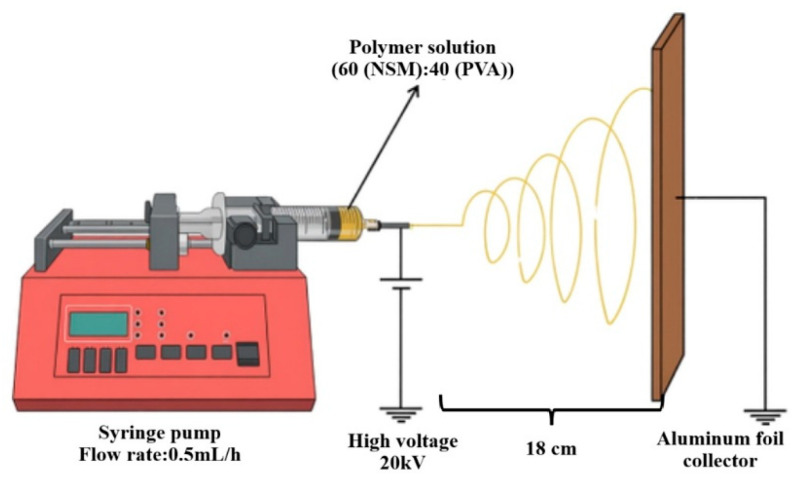
Schematic view of the electrospinning method.

The electrospinning device (NL-MS/K, Nanolysis, Ankara, Turkey) consists of a high-voltage current source, a syringe pump, a collector part and a capillary needle tip syringe [20], as schematically illustrated in Figure 2, which shows the syringe pump, high-voltage supply, and grounded aluminum foil collector used to form the NSM/PVA nanofiber mats. NSM and PVA solutions were thoroughly mixed and homogenized. These solutions were put into a 5 mL syringe. After various studies were carried out on the electrospinning device (summarized in Table 1), the most suitable conditions were set as electric potential 20 kV, distance between the needle tip and the collector 18 cm and solution flow rate 0.5 mL/h. The produced NSM/PVA nanofibers were collected on the aluminum foil, carefully peeled off, and stored in sealed polyethylene bags at room temperature (~22–25 °C) in a desiccator to protect the hygroscopic mucilage-containing fibers from ambient moisture until characterization.

For nanofiber production, 10% PVA and 2% NSM solutions were mixed in the ratios of 20:80, 40:60, 50:50, 60:40 and 80:20. A total of 5 mL of each formulation was prepared and transferred into a 5 mL syringe. For the optimized 60:40 formulation, 3.0 mL of NSM solution was mixed with 2 mL of PVA solution. In addition, electrical potential studies between 18 and 24 kV, 12–20 cm distance between the needle tip and the collector, and 0.1–2 mL/h solution flow rate were also carried out.

The different NSM/PVA Formulations were initially prepared to optimize the electrospinning process. The formulations were evaluated according to their electrospinnability, fiber continuity, bead formation, and overall fiber morphology. Therefore, only this optimized formulation was selected for detailed physicochemical characterization (SEM, FTIR, XRD, and antioxidant analysis).

### 2.3. Characterization of Nanofibers

Scanning electron microscopy (FEI Nova Nanosem 650, FEI Company, Hillsboro, OR, USA) was used to examine the structure and diameters of NSM-PVA nanofibers. The electrospun nanofibers were collected on aluminum foil and subsequently coated with a thin gold-palladium layer using a sputter coater prior to SEM analysis. The mean diameter of 20 random fibers was measured by image analysis using ImageJ software (Version 1.54, National Institutes of Health, Bethesda, MD, USA) from different SEM images. Fourier transform infrared spectrophotometer (Bruker Alpha, Bruker Optik GmbH, Ettlingen, Germany) was used to study the chemical interactions of NSM-PVA nanofibers. The spectra have a resolution of 4 cm^−1^ and a wavelength of 400–4000 cm^−1^. X-ray diffraction (PANalytical Empyrean, Malvern Panalytical, Almelo, The Netherlands) patterns were performed to analyze the crystal structures of NSM-PVA nanofibers. Analyzes were performed with Cu Kα radiation (λ = 0.154 nm), 40 kV voltage, and a step size of 1°/min in the range of 2θ = 10–100°.

### 2.4. Determination of Antioxidant Activity

In determining antioxidant activity, the use of radical DPPH is common. The reason for the popularity of this method is its simplicity and high sensitivity [21]. Then, 0.2 mM 1,1-Diphenyl-2-picrylhydrazyl (DPPH) radical solution was prepared with methanol. Nettle seed mucilage prepared with different concentrations (500, 750, 1000, 1500 and 2000 μg/mL) with ultrapure water was added to 2 mL of 0.2 mM DPPH solution. After mixing, the sample was placed in a dark room at 25 °C for 30 min and then the absorbance at 517 nm wavelength was measured. The control contained water instead of nettle seed mucilage solution, while the blanks contained methanol instead of DPPH solution. The following Equation (1) was used to determine antioxidant activity. Additionally, absorbance values in the range of 250–800 nm were measured with a UV-Vis spectrophotometer (Perkin Elmer Lambda 750, PerkinElmer Inc., Waltham, MA, USA).

% DPPH radical scavenging activity = [1 − (ABS (sample) − ABS (blank))/ABS (control)] × 100, (1)

where ABS (sample) is the absorbance of the DPPH solution mixed with the NSM sample, ABS (blank) is the absorbance of the NSM sample mixed with methanol instead of DPPH solution (to correct for the sample’s own color/absorbance), and ABS (control) is the absorbance of the DPPH solution mixed with water instead of the NSM sample.

## 3. Results and Discussion

### 3.1. Morphological Analysis of NSM and NSM-PVA Nanofibers by SEM

**Figure 3:**
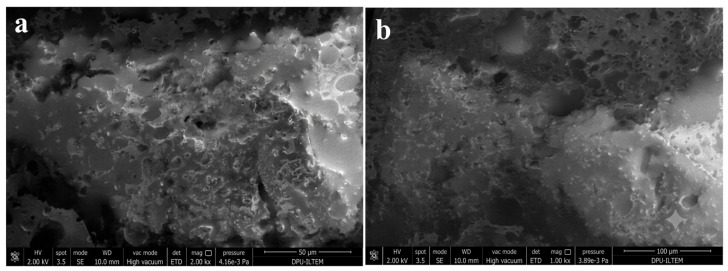
SEM images of NSM at (**a**) 2 × 10^3^ and (**b**) 1 × 10^3^ magnification, showing the particulate, smooth-surfaced morphology of the dried mucilage powder.

SEM images of stinging nettle seed mucilage are given in Figure 3. It has been observed that this structure has a regular morphology. The reason why the structure is smooth and in particles is the polysaccharide structure of the mucilage. This polysaccharide structure is also compatible with FTIR studies.

Electrospinning is a method that can produce fibers in the nanometer–micrometer range, providing network structures with high specific surface area and high porosity. PVA-based electrospun matrices are being extensively studied, particularly in biomedical applications, wound dressings, drug delivery, and tissue engineering. Fiber diameter and morphology are sensitive to numerous parameters, including solution viscosity, conductivity, polymer concentration, flow rate, needle-collector distance, and environmental conditions [22].

SEM was used to examine the morphology of NSM-PVA nanofibers and to determine the diameters of the nanofibers. Although all NSM/PVA formulations were electrospun during the optimization stage, detailed SEM characterization was performed only for the optimized 60:40 formulation. The remaining formulations were used solely to determine the most suitable electrospinning conditions and were not subjected to further structural characterization. When the SEM images of the nanofibers were examined, it was determined that the most uniform structure was 60:40 mixing ratio, 20 kV electric potential, 18 cm distance between the needle tip and the collector, and 0.5 mL/h solution flow rate.

**Figure 4:**
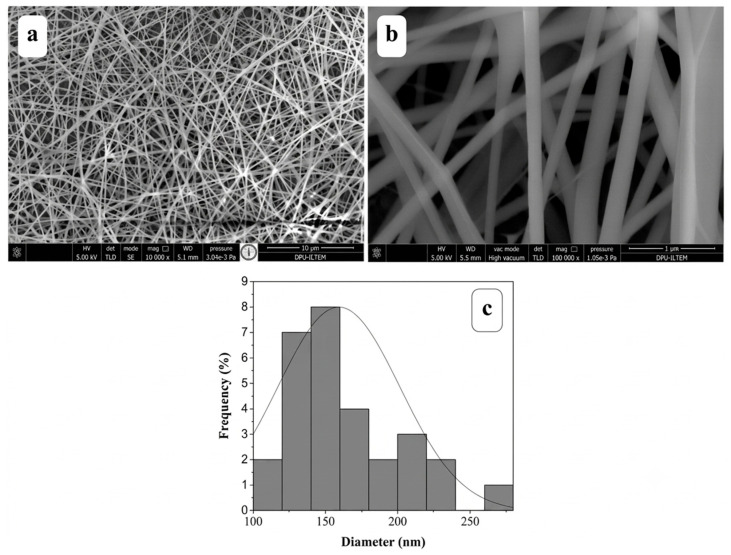
SEM images of NSM/PVA nanofiber at (**a**) 1 × 10^4^ and (**b**) 1 × 10^5^ magnification and (**c**) Histogram of diameter distribution of nanofibers.

SEM images and diameter distribution of the produced nanofibers are given in Figure 4. SEM analyses revealed that the electrospun NSM/PVA nanofiber mat has a porous network structure consisting of randomly oriented, continuous fibers without significant bead defects. It was observed that the fiber surfaces are generally smooth and the fibers exhibit a homogeneous distribution at the nanometric scale. As a result of fiber diameter measurements performed using ImageJ software (NIH, USA) on 20 randomly selected points from SEM images, the average fiber diameter was determined to be 159.26 ± 22.0 nm. The diameter distribution histogram showed that the majority of the fibers are clustered in the approximately 120–170 nm range. These results suggest that the NSM/PVA solution offers a suitable spinnability window for electrospinning and that the jet stability is sufficient.

Compared with the literature, the average diameter obtained is lower than the values reported for basil seed mucilage/PVA (179–390 nm) [9] and Plantago major seed mucilage/PVA (250 nm) [23], and is quite consistent with the values reported for cress seed mucilage/PVA (90.25–169.95 nm) [24] and cactus mucilage/PVA (158 ± 18 nm) [10]. In this respect, NSM/PVA nanofibers exhibit a fine fibrous and competitive morphology among PVA-based natural polysaccharide nanofibers (Table 2).

The fibers had an average diameter of 159.26 nm, which is considered advantageous fort he intended application. Electrospun structures stand out in applications requiring transport, release, and surface interaction due to their high porosity and large surface-to-volume ratio. Therefore, the current morphology appears structurally promising for biomedical coatings, controlled release systems, filtration, or applications requiring active surfaces.

### 3.2. Chemical Structure Analysis of NSM, PVA and NSM/PVA Nanofibers by FTIR

**Figure 5:**
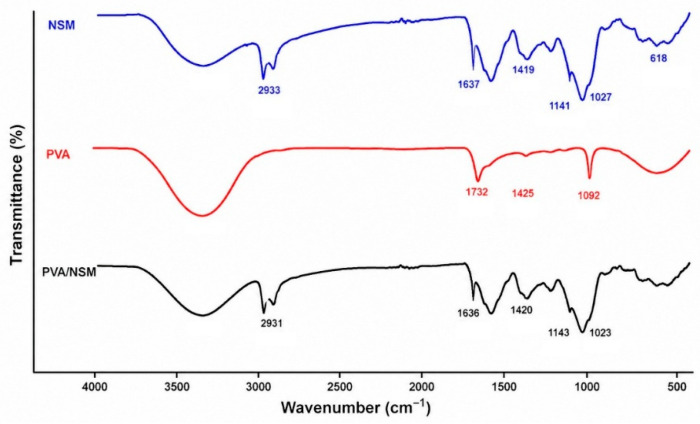
FTIR spectrums of NSM, PVA, NSM-PVA nanofibers.

FTIR analysis was carried out only on the optimized NSM/PVA (60.40) nanofiber, since the other formulations were prepared exclusively for optimization of the electrospinning conditions. According to the FTIR spectrum given in Figure 5, the 1732 cm^−1^ band observed in the PVA spectrum shows the C=O vibrations of residual acetate groups in partially hydrolyzed PVA; the 1425 cm^−1^ band shows CH_2_ bending; and the 1092 cm^−1^ band shows C–O stretching vibrations [29]. This FTIR profile is consistent with the PVA bands described in the literature and reveals that the PVA used exhibits a structure rich in hydrogen bonds and containing trace levels of acetate units. In the literature, it has been stated that the band around 1141 cm^−1^ is sensitive to the crystalline phase [30].

FTIR analyses have shown that NSM exhibits a typical polysaccharide structure. In the NSM spectrum, the broad band in the 3200–3500 cm^−1^ region can be attributed to hydroxyl groups, the 2933 cm^−1^ band to aliphatic C–H stretching, the 1637 cm^−1^ band to uronic acid-derived carboxylate/protein-doped amide I and/or water-bound vibrations, the 1419 cm^−1^ band to C–OH/CH_2_ deformations, and the 1141 and 1027 cm^−1^ bands to C–O–C/C–OH vibrations belonging to the polysaccharide backbone [17]. These assignments are consistent with other studies reporting the presence of carboxyl and hydroxyl groups with glycosidic bonds in nettle seed gum and describing it as a seed hydrocolloid with high uronic acid content and acidic character [16,17,31,32].

The preservation of the 2931, 1636, 1420, 1143, and 1023 cm^−1^ bands in the NSM/PVA spectrum indicates the presence of functional groups belonging to both NSM and PVA in the system. However, the absence of the 1732 cm^−1^ band in pure PVA in the mixture spectrum and the observation of only limited shifts in the existing bands suggest that the mixture forms a compatible structure stabilized by interpolymer hydrogen bonds rather than a new covalent bond formation. In particular, it appears that the interactions between the –OH groups of PVA and the –OH and COO- groups of NSM cause spectral redistribution. Similar FTIR behavior has been reported in different PVA/polysaccharide systems; in some studies, the interaction has been interpreted as strong homogenization, while in others it has been interpreted as a physical mixture that does not form a chemical reaction. The spectral pattern obtained in this study is closer to the physical compatibility model based on hydrogen bonding [33].

When this FTIR spectrum is evaluated together, the NSM/PVA mixture appears to be a compatible physical mixture balanced by hydrogen bonds rather than the formation of a new covalent bond. This is because no new diagnostic band was formed in the spectrum; instead, small shifts in the existing bands and weakening in the PVA carbonyl band were observed. Therefore, it can be said that the spectrum shows successful miscibility and intermolecular interaction between NSM and PVA, and that this interaction occurs through non-covalent/hydrogen bonds [17].

### 3.3. Crystalline Structure Analysis of NSM, PVA and NSM/PVA Nanofibers by XRD

**Figure 6:**
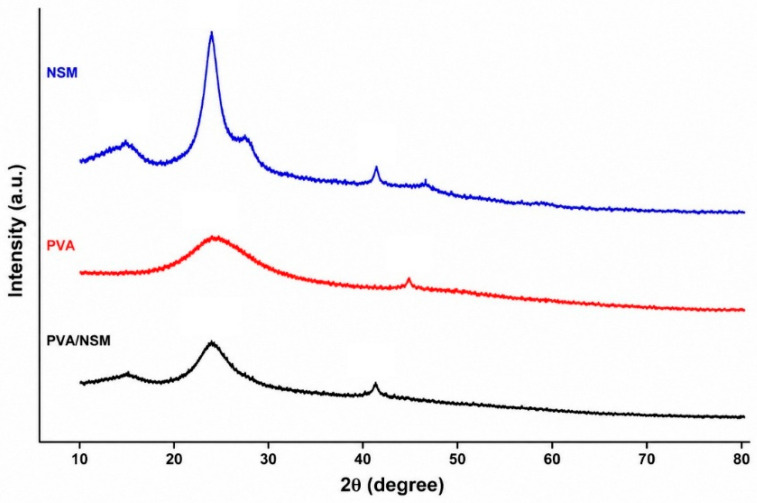
XRD patterns of NSM, PVA and NSM-PVA nanofiber.

XRD characterization was performed only for the optimized 60:40 NSM/PVA nanofiber to evaluate its crystalline structure after optimization of the electrospinning parameters. Figure 6 shows the XRD patterns of NSM, PVA, and NSM/PVA nanofiber.

When the XRD patterns are examined, a very distinct and narrow main peak around ~19°, a secondary contribution around ~23°, and a weak high-angle peak around ~41° are seen in the PVA sample. The PVA pattern in the graph is quite consistent with the classical semi-crystalline PVA behavior. The most dominant feature is the main peak around ~19°; this peak has been regularly reported in the literature as the characteristic reflection of PVA [34].

Instead of distinct narrow peaks in NSM, a broad peak around ~19° was observed. This type of broad band suggests that the long-range crystalline order is weak, i.e., it has acquired a more amorphous or poorly ordered polysaccharide/gum structure. This amorphous behavior has also been clearly reported in published studies on nettle seed mucilage. The hydro-colloidal nature of nettle seed mucilage tends to give broad peaks rather than sharp crystalline peaks thanks to its high carbohydrate and uronic acid content [16].

The PVA/NSM curve shows a profile between pure PVA and pure NSM, but closer to the amorphous character of NSM. The ~19° peak is broadened, the ~23° peak in PVA is largely lost, and the weak PVA peak around ~41° is suppressed. These results indicate that NSM restricts the growth and ordered packing of PVA crystallites, thus resulting in a more amorphous structure of the composite nanofiber [35].

The regular crystal packing of PVA is disrupted after the addition of NSM and electrospinning. Similarly, in the study by Sen et al., they reported that the characteristic sharp peaks around 19.5° and 40° for pure PVA disappeared with the addition of hibiscus leaf mucilage and pectin, and the crystallinity decreased [36]. Another study showed that while the peak persists around 19.5° in PVA/chitosan-based electrospun nanofibers, the broadening and weakening of the peaks indicates a decrease in crystallites in solution and a more amorphous structure [37].

XRD results revealed that while pure PVA exhibited a semi-crystalline structure, NSM showed an amorphous character; in the electrospun PVA/NSM nanofiber, the characteristic peak of PVA around 19.5° broadened and shifted to a lower intensity, while the crystalline additions around 22–23° and 40° were largely suppressed. This indicates that the interactions between NSM and PVA and the electrospinning process disrupted the PVA crystalline structure, resulting in a more amorphous nanofiber. NSM acted not as a filler that preserves the semi-crystalline arrangement of PVA, but as a natural biopolymer component that suppresses it, broadens the peaks and makes the composite more amorphous. This result is a feature often desired, especially in areas such as wound dressings, controlled release and biodegradable carrier systems. This is because more amorphous nanofiber mats can often be associated with higher swelling, faster dissolution/degradation or more homogeneous active substance distribution [38].

### 3.4. Antioxidant Activity of NSM

In this study, the antioxidant activity of nettle seed mucilage was determined by DPPH radical scavenging test. The experiments were performed in three replicates. Accordingly, the reduction rate of 2 mL of 0.2 mM DPPH radicals by 2 g of nettle seed mucilage (NSM) was determined to be 69.76 ± 1.48%. In this case, the average antioxidant activity of nettle seed mucilage was found to be 30.24%. Zamani et al. [17] reported that the antioxidant activity of Nettle seed (*Urtica pilulifera*) gum at a concentration of 1000 μg/mL was 38.1%. It shows that mucilages obtained from two Nettle seed species (*Urtica dioica* and *Urtica pilulifera*) have various compositions.

**Figure 7:**
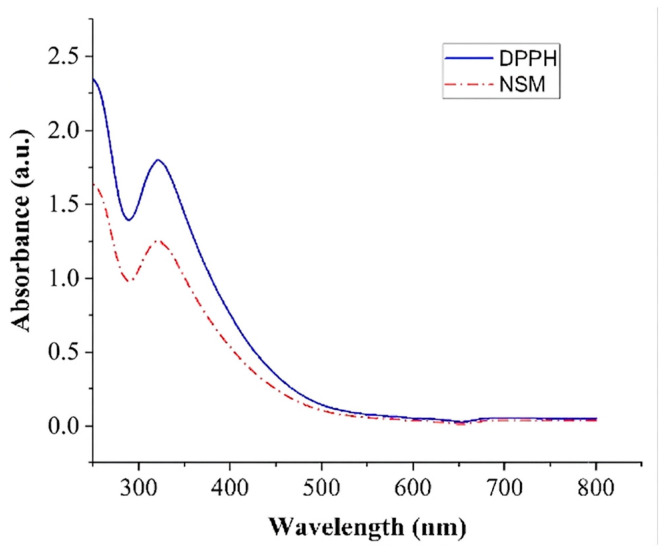
UV-Vis spectrum showing the DPPH radical scavenging activity of 0.2 mM DPPH and NSM.

The UV-Vis spectra of the DPPH radical scavenging amount of nettle seed mucilage are shown in Figure 7 below.

Findings from some similar studies in the literature are presented in Table 3.

## 4. Conclusions

This study demonstrates that nettle seed mucilage can be successfully converted into nanofiber form using electrospinning with PVA. Among different mixing ratios and production conditions, the most suitable nanofiber morphology was obtained. SEM images revealed that the produced nanofibers were uniform, continuous, bead-free, and homogeneously distributed; the average fiber diameter of 159.26 nm indicated that the system is advantageous for applications requiring high surface area. FTIR analyses showed that NSM and PVA coexist in the structure and that physical interactions based on hydrogen bonds occur between the two components. The absence of a new characteristic band supports the idea that a compatible polymer–biopolymer mixture, rather than covalent bonding, is formed in the system. XRD results showed that NSM partially disrupted the crystalline structure of PVA and made the nanofiber matrix more amorphous. This finding can be considered particularly favorable for biomedical and controlled-release applications where properties such as swelling, dissolution, biodegradability, and active substance distribution are important. Furthermore, the DPPH test revealed that nettle seed mucilage possesses antioxidant activity and can provide functional contributions to the nanofiber system. Overall, the findings indicate that NSM/PVA composite nanofibers represent a potential structure for development of naturally derived, biocompatible, and functional materials. Further research is recommended to investigate these nanofibers in more detail regarding mechanical strength, water stability, biodegradability, cellular compatibility, and controlled-release performance.

## Figures and Tables

**Table 1:** Electrospinning parameter trials examined for NSM/PVA nanofiber optimization in this study.

Trial No.	NSM:PVA Ratio	NSM (mL)	PVA (mL)	Voltage (kV)	Distance (cm)	Flow Rate (mL/h)
1	20:80	1	4	18–24	12–20	0.1–2
2	40:60	2	3	18–24	12–20	0.1–2
3	50:50	2.5	2.5	18–24	12–20	0.1–2
**4**	**60:40**	**3**	**2**	**20**	**18**	**0.5**
5	80:20	4	1	18–24	12–20	0.1–2

**Table 2:** Some comparative data about the average diameter of various NSM/PVA nanofibers.

Nanofiber Material	Ratio	Diameter (nm)	Fiber Morphology	Reference
NSM/PVA	60:40	159.26	Visually smooth, continuous, bead-free nanofibers.	This study
Rocket seed mucilage/PVA	60:40	102.3	Beadless, uniform, and smooth nanofibers.	[11]
Flaxseed mucilage/PVA	60:40	123.46 ± 2	Uniform, bead-free nanofibers.	[25]
Alyssum Lepidium mucilage/PVA	80:20	139.9	Obtain uniform nanofibers with low diameters.	[26]
Basil seed mucilage/PVA	60:40	179–390	Increasing the proportion of BSM in the solution results in finer fibers, while increasing the proportion of PVA results in smoother, bead-free nanofibers.	[9]
Plantago major seed mucilage/PVA	50:50	250	Smooth, bead-free nanofibers.	[23]
Cress seed mucilage/PVA	60:40	90–169	Smooth and homogeneous nanofibers.	[24]
Sage seed mucilage/PVA	70:30, 60:40, 50:50	130–300	Beadless, 50:50 nanofibers have a more irregular, fractured, and variable diameter.	[27]
Cactus mucilage/PVA	80:20	158 ± 18	Continuous and uniform nanofibers.	[10]
Chia seed mucilage/PVA	60:40	72	Smooth, bead-free, homogeneous nanofibers.	[28]

**Table 3:** Some studies have been conducted on the antioxidant properties of *Urtica dioica* plant.

Sample Type	Extraction/Matrix	DPPH Result	Brief Commentary	Reference
*Urtica dioica* aerial parts	Ultrasonic bath 80% ethanol; boiling with water; fermentation with water.	IC50 6.20/13.77/10.02 mg/mL	Strongest antioxidant profile in ultrasonic ethanol extract.	[39]
Dried leaf	Optimized water extraction, ultrasound.	86.6%	High activity level; water + ultrasound combination is the determining factor.	[40]
*Urtica dioica* extract	Methanol, hexane, water	62.42%	The methanol extract is the most potent in DPPH.	[41]
*Urtica dioica* powder	Methanol extract	71.98%	One of the closest values to NSM	[42]
Cake filtrate with 5% nettle seeds	Aqueous supernatant	33.8%	Lower than expected due to the food matrix.	[43]
Leaf and flower polyphenolic fractions	Accelerated extraction + SPE purification	IC50 78.56/124.77 µg/mL	Leaf fraction is more potent than flower fraction	[44]
*Urtica dioica* extract	Acidified methanol and hexane	84.36 ± 1.50 mg TE/g	The phenolic-rich methanolic fraction is dominant	[45]
Nettle seed gum analog	*Urtica pilulifera* gum	%38.1 (1000 µg/mL)	More limited DPPH can be expected in pure/semi-pure gum systems.	[17]

## Data Availability

The raw data supporting the conclusions of this article will be made available by the authors on request.

## Abbreviations

The following abbreviations are used in this manuscript:

NSM	Nettle seed mucilage
PVA	Polyvinyl alcohol
